# Prevalence and Associated Risk Factors of Age-Related Macular Degeneration in the Retina Clinic at a Tertiary Center in Makkah Province, Saudi Arabia: A Retrospective Record Review

**DOI:** 10.7759/cureus.36048

**Published:** 2023-03-12

**Authors:** Fatmah H Abusharkh, Layan Kurdi, Rahaf W Shigdar, Rahaf A Mandura, Khadija Alattas

**Affiliations:** 1 Department of Medicine, King Abdulaziz University Faculty of Medicine, Jeddah, SAU; 2 Department of Ophthalmology, King Abdulaziz University Hospital, Jeddah, SAU

**Keywords:** age, smoking, epidemiology, retinal disorder, retrospective record review

## Abstract

Background

Age-related macular degeneration (AMD) is a progressive disease involving the macular region of the retina and is considered a significant cause of vision loss worldwide. With the increase in life expectancy in various countries, this problem has become more apparent. We retrospectively evaluated the prevalence of AMD among patients visiting the retina clinic at King Abdulaziz University Hospital (Jeddah, Makkah Province, Saudi Arabia) to identify the commonly associated risk factors of AMD.

Methods

The records of 3,067 individuals from 2017 to 2021 were reviewed. Of these, 1,935 were enrolled in the study.

Results

The prevalence of AMD among the patients was 4%. Regarding non-modifiable risk factors, age and having a family history of AMD showed a significant association (p = 0.001 and 0.043, respectively). However, sex and nationality were not significantly associated. As for modifiable risk factors, smoking and hypertension demonstrated a significant relationship (p < 0.001 and p = 0.002, respectively). However, the association with diabetes mellitus and dyslipidemia was not significant.

Conclusion

Our study shows that AMD is widely prevalent in Saudi Arabia and is associated with age, family history, smoking, and hypertension. Therefore, patients at risk of AMD must be screened and managed promptly before disease progression.

## Introduction

Age-related eye disorders in older individuals are serious health issues that severely impact the quality of life of millions globally. The severity of this problem has become more obvious due to the increase in life expectancy in various countries [1]. Cataracts, age-related macular degeneration (AMD), glaucoma, and diabetic retinopathy (DR) are the main age-related eye illnesses [2]. In 2020, the fourth leading cause of blindness worldwide was AMD [3]. The global prevalence of AMD is estimated to be around 8.7% [4]. By 2040, global demographic shifts are expected to increase the number of AMD patients to 288 million [4].

AMD is a progressive neurodegenerative disease affecting the macula [5]. The pathophysiology of AMD is complex and includes major abnormalities in four interrelated tissues: the choriocapillaris, Bruch's membrane, retinal pigment epithelium (RPE), and photoreceptors [6]. The impedance of RPE cell function is a crucial step in the molecular pathways leading to clinically significant AMD changes [6]. Irreversible degeneration of photoreceptors is caused by RPE degeneration [6]. Yellowish lipid-rich, protein-containing drusen deposits accumulate between Bruch's membrane and the RPE [7,8]. Furthermore, lipofuscinogenesis, drusogenesis, inflammation, and neovascularization are four main processes responsible for the formation of the two types of AMD: the wet (exudative, neovascular) and dry (non-exudative, geographic atrophy) types [6].

Various studies have assessed AMD prevalence in different countries. In 2014, a meta-analysis of 39 studies, including 129,664 individuals, found that the prevalence of AMD was 8.69% [4]. A retrospective study conducted in Spain among 119,877 individuals showed that the prevalence of AMD was 7.6% [9]. Slightly lower percentages were found in Korea and Germany, 6.62% and 3.3% respectively [10,11]. A cross-sectional study performed in the United Kingdom (2017) concluded that among the Caucasian population aged 65 years and older, 1.8% had wet AMD and 2.5% had dry AMD [12]. Locally, in Saudi Arabia, a study assessing the various causes of blindness found that 3.3% of cases were attributed to AMD [1]. Regarding the risk factors, several factors were linked to the development of AMD. Age and genetic background were identified as strong non-modifiable risk factors [1]. Another non-modifiable but inconsistent risk factor was gender [1]. Among the modifiable risk factors such as smoking, cardiovascular disease, hypertension, diabetes mellitus, and dyslipidemia, smoking was shown to be the main factor [1].

Only a handful of studies on AMD have been conducted in Saudi Arabia, and notably, there is a dearth of research - especially in the western region. Moreover, further data regarding risk factors are required to emphasize potential associations. Therefore, this study aimed to evaluate the prevalence of AMD among patients visiting the retina clinic at King Abdulaziz University Hospital (KAUH), Jeddah, Saudi Arabia, and to assess the commonly associated risk factors for AMD.

## Materials and methods

A retrospective review of the records of 3,067 outpatients who attended the Retina Clinic at the Department of Ophthalmology at the KAUH in Jeddah, Saudi Arabia, from 2017-2021 was conducted. Of these, 1,935 satisfied the inclusion criteria and were included in this study. The study design was approved by the Research Ethics Committee of King Abdulaziz University (KAU) with approval number 398-21 on August 10, 2021.

Participants

The medical records of patients aged ≥18 years were reviewed between 2021-2022 to ascertain the prevalence and frequently related risk factors for AMD. Patients below 18 years and those who did not undergo fundus imaging, or attend their booked appointments, follow-ups, treatments, and referrals were excluded.

Data collection

Patients' data, including their demographic data - such as age, sex, and nationality - and clinical data, which included their diagnosis, smoking status, family history, and medical history, were retrieved from the hospital's database (Phoenix). For those diagnosed with AMD, further information was collected, including the type of AMD, date and duration of diagnosis, and affected eye. Information not found on the hospital's database, such as smoking status and family history, was obtained by contacting the patients through mobile phones.

Ophthalmological examination

Participants underwent non-mydriatic fundus photography performed by trained and certified optometrists. AMD was diagnosed based on optical coherence tomography (model 2000; Zeiss-Humphrey Instrument Inc., San Leandro, CA). All examinations were conducted using standardized operational procedures, and data were electronically recorded.

Statistical analysis

A data collection sheet was created using Google Forms, and the data obtained were then transferred to Microsoft Excel 2016 (Microsoft, Redmond, WA). Data were interpreted using SPSS version 26 (IBM Corp., Armonk, NY). Categorical data were expressed as frequencies and percentages, whereas continuous variables were described as arithmetic means and standard deviations. Chi-squared or Fisher's exact tests were used to investigate the association between two categorical variables. In contrast, the independent t-test was used to compare the means of continuous variables between two different groups. Statistical significance was set as p < 0.05.

## Results

This study included 1,935 patients who attended the retina clinic at KAUH, Saudi Arabia.

Prevalence and determinants of AMD

**Figure 1:**
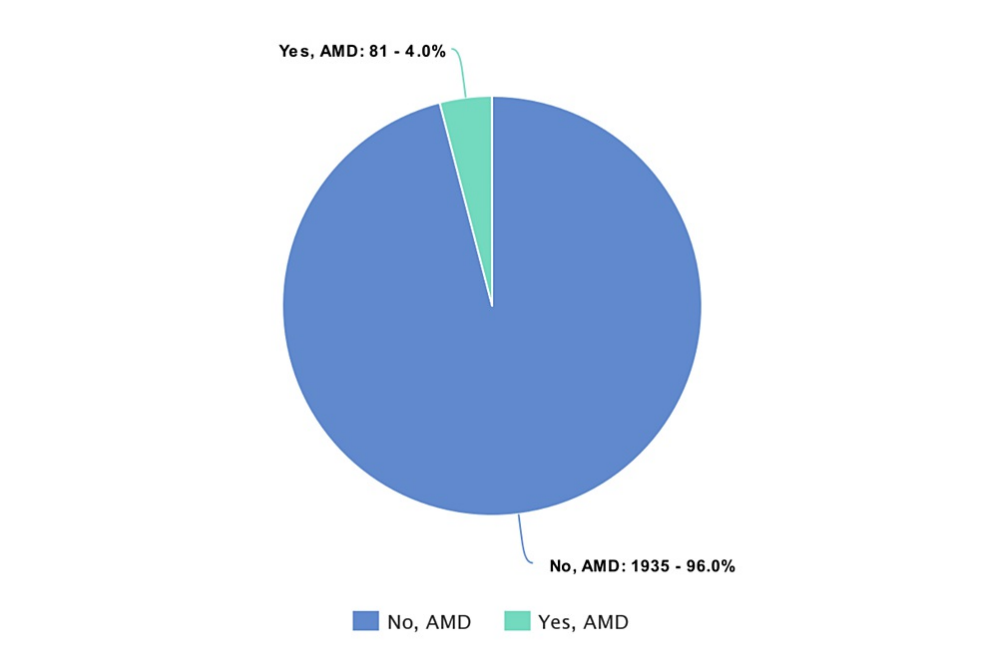
Prevalence of age-related macular degeneration (AMD) among patients attending the retina clinic at King Abdulaziz University Hospital, Jeddah (2017–2021).

As illustrated in Figure 1, the prevalence of AMD among patients attending the ophthalmology clinic at KAUH between 2017 and 2021 was 4%. In Table 1, it is shown that the age of patients with AMD was significantly greater than those without AMD (72.4 ± 9.8 years vs. 57.2 ± 15.5 years; p < 0.001). Family history and smoking status were successfully tracked in only 118 and 662 patients respectively, while the remainder were unattainable or unknown. Participants with a family history of AMD tended to have the disease more than those without such a history (85.7% vs. 45%; p = 0.043). Ex- and current smokers were more likely to have AMD than non-smokers (34% and 18.6% vs. 7.2%; p < 0.001). Patients with hypertension and those without type 1 diabetes were at a higher risk of developing AMD than those without hypertension (5.5% vs. 2.8%; p = 0.002) and those with type 1 diabetes (4.2% vs. 0.8%; p = 0.040). In contrast, sex, nationality, type 2 diabetes, and abnormal lipid profile were not significantly associated with AMD.

**Table 1:** Risk factors for AMD among patients attending the retina clinic AMD: Age-related macular degeneration

		AMD		p-value
		Yes N=81	No N=1935	
Age Mean±SD		72.4±9.8	57.2±15.5	<0.001*
Sex	Male, N (%)	43 (4.9%)	827 (95.1%)	0.065^†^
	Female, N (%)	38 (3.3%)	1108 (96.7%)	
Nationality	Saudi, N (%)	46 (3.6%)	1238 (96.4%)	0.187**
	Non-Saudi, N (%)	35 (4.8%)	697 (95.2%)	
Family history of AMD (n=118)	N=118	N=56	N=62	0.043^‡^
	No, N (%)	50 (45.0%)	61 (55.0%)	
	Yes, N (%)	6 (85.7%)	1 (14.3%)	
Smoking status (n=662)	N=662	N=65	N=597	<0.001^†^
	Never, N (%)	41 (7.2%)	531 (92.8%)	
	Current, N (%)	8 (18.6%)	35 (81.4%)	
	Ex-smoker [5-10 years], N (%)	16 (34.0%)	31 (66.0%)	
Hypertension	No, N (%)	31 (2.8%)	1077 (97.2%)	0.002^†^
	Yes, N (%)	50 (5.5%)	857 (95.4%)	
Diabetes mellitus Type 2	No, N (%)	25 (3.8%)	636 (96.2%)	0.702^†^
	Yes, N (%)	56 (4.1%)	1297 (95.9%)	
Diabetes mellitus Type 1	No, N (%)	80 (4.2%)	1814 (95.8%)	0.040˚
	Yes, N (%)	1 (0.8%)	119 (99.2%)	
Dyslipidemia	No, N (%)	66 (3.9%)	1642 (96.1%)	0.395^†^
	Yes, N (%)	15 (4.9%)	291 (95.1%)	

Clinical characteristics of AMD

**Figure 2:**
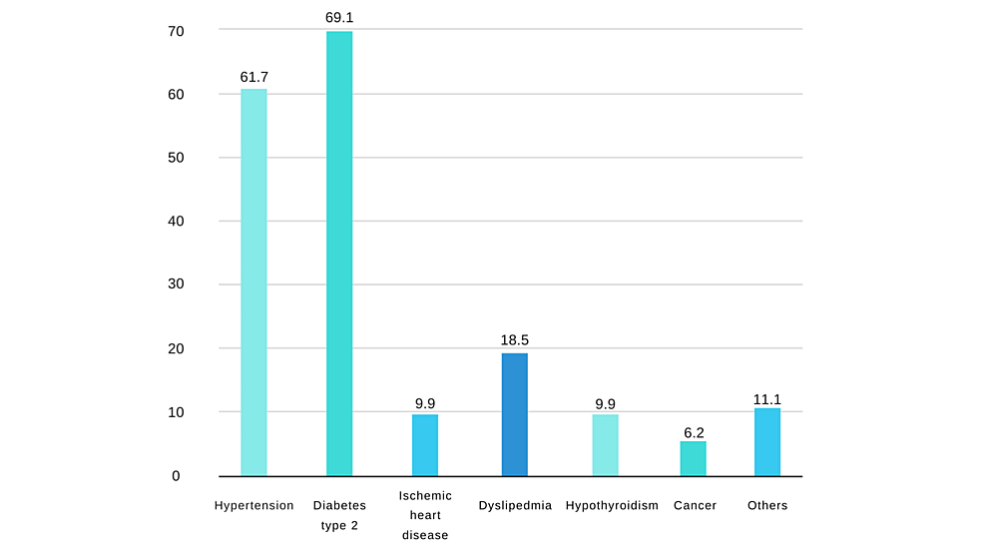
Chronic medical problems associated with age-related macular degeneration.

Table 2 summarizes the clinical characteristics of AMD cases. Most cases (70.4%) were of the dry type and affected both eyes (77.2%). The disease duration was ≥5 years in 43.1% of the patients. The most frequent chronic diseases associated with AMD were type 2 diabetes (69.1%), hypertension (61.7%), and dyslipidemia (18.5%), as shown in Figure 2.

**Table 2:** Clinical characteristics of AMD cases, retina clinic, King Abdulaziz University Hospital, Jeddah (2017–2021) AMD: Age-related macular degeneration

		Frequency	Percentage
Diagnostic type	Dry	57	70.4
	Wet	18	22.2
	Both	6	7.4
Affected eye (n=79)	Unilateral	18	22.8
	Bilateral	61	77.2
Duration (years) (n=72)	<5	41	56.9
	≥5	31	43.1

## Discussion

Our study results revealed that the prevalence of AMD in the Retina Clinic at the Department of Ophthalmology at the KAUH in Jeddah, Saudi Arabia is 4%, with older age, smoking, family history, and hypertension being the most common significant risk factors associated with AMD. A meta-analysis of 39 population-based studies found that AMD was associated with an overall prevalence of 8.69% [4]. Moreover, according to a retrospective study review conducted in Spain of 8,504 participants, AMD was present in 7.6% of the population [9]. In addition, cross-sectional studies conducted in Egypt and Korea found AMD to be similarly prevalent, at 6.6% and 6.62%, respectively [10,13]. Furthermore, among 8,504 participants in an Irish cross-sectional study in 2015, the prevalence of AMD was estimated to be 7.2% [14]. Compared to our study, most other studies showed a higher prevalence due to differences in the sample size and study design.

Most patients in our study had dry AMD, followed by wet AMD, and the rest had both. Similarly, a Spanish study found that dry AMD is more prevalent than wet AMD [9]. However, the prevalence of wet AMD is twice as high as that of dry AMD in some other studies [15,16].

In our study, 22.8% of patients had AMD in one eye, while most had bilateral AMD. Wang et al. concluded that among all cases diagnosed with AMD, 57% had bilateral involvement [17]. Likewise, in a Spanish study, most patients had bilateral AMD [9]. Furthermore, the Australian National Eye Health Survey found that 42.9% of 1,141 individuals diagnosed with AMD had bilateral involvement [18]. Additionally, it was observed that even patients initially diagnosed with AMD in one eye were eventually affected in both eyes [18]. This could be due to the progressive course of the disease and the lack of follow-up and poor commitment to the treatment plan [18].

Non-modifiable risk factors

The mean age of AMD patients in this study was 72.4 ± 9.8 years compared to non-affected patients, which was 57.2 ± 15.5 years. Advanced age demonstrated the most significant association with AMD (p < 0.001). Many studies in the literature are consistent with this result [1,4,9,10,15,19].

Further, a significant association was found between family history and AMD (p = 0.043). Compared with the general population, siblings of those diagnosed with AMD have a three to six times greater risk of developing the disease [20]. Moreover, in the Al-Khobar study, family history was consistently associated with AMD incidence [1]. Smith and Mitchell discovered a significant relationship between family history and AMD, with the association being strongest in cases of wet AMD [21]. Similarly, a population-based cohort with a four-year follow-up from the ALIENOR study found a significant association between genetic background and AMD [22]. Overall, most studies have shown a positive association with family history due to the genetic predisposition to the disease [23].

Most studies showed weak and inconsistent associations with sex [1,4,10,11,15,24]. Similarly, our study showed that AMD is more prevalent in males; nonetheless, this association was not significant (p = 0.065). However, in an Egyptian study, male participants had a prevalence of AMD of 9.2%, while female participants only had a prevalence of 4.1%; this difference was statistically significant (p = 0.017) [13]. On the contrary, some other studies have reported that females had a higher prevalence of AMD [9, 23, 25].

Modifiable risk factors

This study showed that ex-smokers have the greatest chance of developing AMD. Nonetheless, both ex-smokers and smokers had a significantly increased AMD prevalence (p = 0.001) [23]. Multiple studies, including the Rotterdam Study, have discovered a significant association between AMD and smoking [1,25-29]. This association showed a particularly strong progression of AMD in younger age groups (<85 years), which was demonstrated in current smokers and ex-smokers compared to non-smokers [26,27]. A cohort study demonstrated a possibility of a four- to six-fold increase in disease advancement and showed that those who never smoked have a higher opportunity to experience regression than current and former smokers [28]. However, the SHIP-Trend population-based study denied a significant link to smoking [11]. It is believed that toxic substances in cigarettes might play a role in the pathological process of AMD by accentuating oxidative stress. Therefore, various studies have proposed smoking as a potential risk factor [23].

Several studies have shown supporting findings indicating a significant relationship between higher blood pressure and the development of AMD [29-32]. These findings are consistent with those of our study (p = 0.002). In contrast, a few studies showed no association between elevated blood pressure and AMD development [12,19,32]. Because both AMD and hypertension are related to older age, the result of our study is biologically plausible.

The most common chronic comorbidity among AMD patients in our study was type 2 diabetes mellitus; however, it was not significantly associated with AMD (p = 0.702). Similarly, they were not consistently associated in most studies [10,12,22]. Based on Spanish and UK studies, diabetes is a protective factor against AMD development [9,32]. In contrast, several studies reported that diabetes and AMD were positively associated [33,34]. This could be justified by the fact that type 2 diabetes, similar to hypertension, is also linked with advanced age, which is the main contributor to AMD. Dyslipidemia was found to be the third comorbidity factor associated with AMD; nevertheless, the relationship was not significant, and most studies have reported no significant association with AMD [11,32,35,36]. Nonetheless, Vassilev et al. observed a slightly elevated chance for the development of AMD among cases of hyperlipidemia [37].

Strength and limitations

This study is the first to explore the prevalence of AMD in Saudi Arabia. Moreover, this study was conducted in one of the leading tertiary centers in the region which included a large sample size. The risk factors assessed in our study were among the most critical potential predictors of AMD development. On the other hand, our study had some limitations. The principal limitation was that it was a retrospective study. As a result, selection and observation biases, in addition to confounding factors, are consequent limitations. The second limitation was the small sample size owing to the inclusion criteria. Thirdly, not all risk factors, such as body mass index, diet, refractive errors, and the presence of other ophthalmological disorders, such as cataracts, glaucoma, and DR, were assessed.

## Conclusions

In summary, our single tertiary center study showed that AMD is widely prevalent in Jeddah, Saudi Arabia (4%) and linked to a wide range of risk factors. Some of these are modifiable risk factors that can be adjusted to help reduce AMD occurrence. Furthermore, this study has shown the importance of screening and follow-up of family members of patients with AMD to promote early detection and intervention of AMD. We recommend conducting further research on AMD in Saudi Arabia. Concerning the study design, a community-based cross-sectional study would be more helpful for assessing the disease's prevalence. Finally, recruiting a larger sample size is required for more accurate estimation.
